# Meeting user needs in national healthcare systems: lessons from early adopter community pharmacists using the electronic prescriptions service

**DOI:** 10.1186/1472-6947-14-16

**Published:** 2014-03-10

**Authors:** Jasmine Harvey, Anthony J Avery, Ralph Hibberd, Nicholas Barber

**Affiliations:** 1 School of Medicine, Division of Primary Care, Queens Medical Centre, University of Nottingham, Nottingham NG7 2UH, UK; 2 Department of Practice and Policy, UCL School of Pharmacy, Mezzanine Floor, BMA House, Tavistock Square, London WC1H 9JP, UK

**Keywords:** User-centric approaches, Healthcare ICT, Usability, User experience, Social informatics in healthcare, Electronic prescription service release two

## Abstract

**Background:**

The Electronic Prescription Service release Two (EPS2) is a new national healthcare information and communication technology in England that aims to deliver effective prescription writing, dispensing and reimbursement service to benefit patients. The aim of the study was to explore initial user experiences of Community Pharmacists (CPs) using EPS2.

**Methods:**

We conducted nonparticipant observations and interviews in eight EPS2 early adopter community pharmacies classified as ‘first-of-type’ in midlands and northern regions in England. We interviewed eight pharmacists and two dispensers in addition to 56 hours recorded nonparticipant observations as field notes. Line-by-line coding and thematic analysis was conducted on the interview transcripts and field notes.

**Results:**

CPs faced two types of challenge. The first was to do with missing electronic prescriptions. This was sometimes very disrupting to work practice, but pharmacists considered it a temporary issue resolvable with minor modifications to the system and user familiarity. The second was to do with long term design-specific issues. Pharmacists could only overcome these by using the system in ways not intended by the developers. Some felt that these issues would not exist had ‘real’ users been involved in the initial development. The issues were: 1) printing out electronic prescriptions (tokens) to dispense from for safe dispensing practices and to free up monitors for other uses, 2) logging all dispensing activities with one user’s Smartcard for convenience and use all human resources in the pharmacy, and, 3) problematic interface causing issues with endorsing prescriptions and claiming reimbursements.

**Conclusions:**

We question if these unintended uses and barriers would have occurred had a more rigorous user-centric principles been applied at the earlier stages of design and implementation of EPS. We conclude that, since modification can occur at the evaluation stage, there is still scope for some of these barriers to be corrected to address the needs, and enhance the experiences, of CPs using the service, and make recommendations on how current challenges could be resolved.

## Background

### The electronic prescription service (Release 2)

In England, it is part of the government’s agenda to enable the writing and dispensing of electronic prescriptions to patients. The Electronic Prescription Service (EPS), released in stages one (EPS1) and two (EPS2) was designed to meet this agenda. Our study focused on EPS2. Sudgen and Wilson [1] who piloted and evaluated early models of the EPS, also known as the Electronic Transmission of Prescriptions, documented the concept behind its development. Rai [2] explains the process of writing and dispensing EPS2 prescriptions in detail, and Gourdrey-Smith [3] explains its functions. In summary, EPS2 is information and communication technology that automates prescription links between prescribing, dispensing and pricing bodies for better patient care in the following simplified format:

• *Patient to General Practitioner (GP)*: GP (or designated prescriber) writes prescriptions electronically.

• *Electronic prescription to Spine*: The electronic prescription is sent to and stored on a national database called the N3, commonly known as the Spine.

• *Spine to community pharmacies*: Community Pharmacists (CPs) and their teams

• (CP professionals) in pharmacies nominated by the patient to dispense their prescriptions can access and download the prescription from the Spine, and dispense it to the patient.

• *Community pharmacies to remuneration agency*: The remuneration agency, formerly known as Prescription Pricing Authority (PPA), is now called NHS Prescription Services. NHS Prescription Services receives prescriptions from dispensers, such as community pharmacies, calculates relevant payments for the items dispensed and remunerates dispensers for these. With EPS2, details of items dispensed are sent directly to NHS Prescription Services electronically.

**Figure 1:**
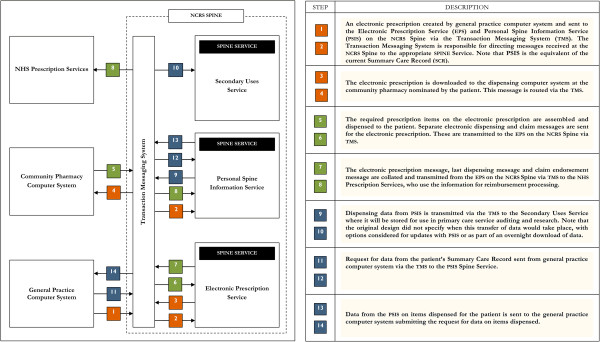
Original EPS2 model and architecture (given to study team by Connecting for Health).

Therefore, prescribers, dispensers, patients and the pricing authority are key stakeholders in the successful implementation of the EPS2. Figure 1 shows the original EPS2 architecture given to the study team by the initial overseeing body called Connecting for Health (CfH). While the wider scope of the research project was to evaluate changes in various forms made by EPS2 to all stakeholders, in this paper we focus on community pharmacies. CP professionals were proposed to be one of the key beneficiaries of the EPS2 for being able to provide a better quality service because of improved work practices. The pharmacist from the first adopter pharmacy began dispensing EPS2 prescriptions in July 2009. Although EPS2 was still undergoing development at the time of data collection (February–September 2011), early adopters were dispensing between 10% and 40% EPS2 prescriptions (Table 1).

**Table 1:** Descriptive information of data collection sites

**Site**	**Percentage of EPS2 dispensed at time of interview**	**EPS2 live since**
1	20%	07.2009
2	35%	12.2009
3	18%	09.2010
4	Information not supplied but noted to be greater than 10%	06.2011
5	40%	08.2010
6	10%	08.2010
7	10%	07.2010
8	16%	05.2010

Since 2011, the National Health Service (NHS) in England, including EPS2 has been undergoing re-organisation. EPS2 was originally part of the National Programme for IT (NPfIT) to make Primary and Secondary care electronically interoperable in England [4], but it was designed to operate independent of other NPfIT programmes [2-5]. When NPfIT was dismantled in 2011, some of its stand-alone programmes such as EPS2 were retained. On 31 March 2013, the overseeing body of the NPfIT programmes CfH ceased to exist. Many of its functions, including the Electronic Prescription Service, have transferred to the new Health and Social Care Information Centre (HSCIC). On 1st April 2013, responsibility for supporting pharmacists in using these services transferred to NHS England (formally known as the NHS Commissioning Board) [6]. The EPS2 national implementation programme is still on going.

The aim of the study was to explore initial user experiences of CP professionals, and to ask if their needs were met concerning the development and implementation of EPS2. In the following, we briefly discuss key factors in new technology adoption and frame our research questions within these contexts. We then discuss our methodology, present and discuss our findings. Please note that we use ‘early adopters’ to denote sites involved in the early stages of EPS2 implementation. Also, note that we use the term ‘real user’ to denote actual users of the system. In this paper, ‘real users’ are practising community pharmacists and their teams, and not any other type of pharmacist.

### Some key factors in successful system delivery or adoption

New systems or innovations are usually developed to solve a problem or aid a process. A system development usually comprises several iterative stages in its lifecycle such as specification or problem-definition, feasibility study, analysis, design, implementation, evaluation and maintenance. When a new system is being developed, many factors influence the planning stage [7]. Key factors include: a) Financial aspects, which comprise of the cost of the system and the overall benefits. b) Technical aspects, what can be accomplished by using existing technologies? and c) Social factors, which comprise changes the new system will be making to current work practices of the users, and how users will engage with the system comprehensively. Some authors recognise this last factor, the social factor, as the most important in a system development concerning the systems acceptance and diffusion [8,9]. Rogers, for example, names five determinants including simplicity and ease of use, and, compatibility with existing values and practices as key factors in successful adoption, and argue that the innovation is usually what changes to suit the user and not the other way round [9]. Many systems fail or fall into disuse not because of technical failure, but in how the technology is matched to the social environment [10]. The disengagement by users, or a poor user experience directly affects financial and technical factors and can render the system unusable and detrimental [11-15]. Consequently, there needs to be a continuous engagement between the system and the social environment to render a product usable [15]. In theorising the role technology plays in social structures, Greenhalgh and Stone [16] explained that, the technological component of social structures may be supported when people choose to use the technology, and, not supported when they actively refuse to use it or, importantly, cannot use it at all or in the ways they would like.

In healthcare, Benn et al. [17] lists “size, regional location, internal structure, management processes, history, external regulatory environment, culture and leadership” as key variables that contribute to successful delivery of care systems. However, similar to other theories, emphasis is placed on social elements as a key factor in technology adoption. Harvey et al. [18] for example conceptualised that “socio-technical interdependence” is a key dimension in the adoption of new technology into pharmacy work practice. Therefore, before new technology is effectively integrated into work practice, it is important to note how the intended users interact with existing technologies in that environment. In their study, which focused on the approaches to, and experiences of user engagement of the adoption of Lorenzo software into national EHR system in England, Cresswell et al. [19] placed emphasis on user (dis)engagement. For example, because real users such as hospital staff were not included in the system development process, this alienated them during the implementation process as they felt the system was then not sufficiently customised for their needs. Furthermore, Gagnon et al. [20] found that the boundary between barriers and facilitators in electronic prescription systems’ adoption were blurred, and recommended studies to be conducted from a variety of user group perspectives. Within these contexts, we frame our research questions: what were CP professionals’ experiences of EPS2, what were their perceptions and attitudes toward the new system, and what can be learned to improve user experience and feedback to the implementers in terms of meeting their needs?

## Methods

### Data collection

The research was part of the national CfH evaluation programme commissioned by Department of Health to evaluate how EPS2 will alter work practices. The research protocol was designed by a multidisciplinary study team consisting of social scientists, academic and practising pharmacists and a general practitioner. The methodological framework was developed from literature reviews on ‘user perception’ studies of new technologies [21-23], electronic prescription adoption in healthcare [24-29] and listed potential benefits of EPS2 according to CfH [30]. Rather than using standardised variables in ‘user perception’ study models such as perceived ease of use and perceived usefulness, we adopted a constructivist (qualitative) approach by using flexible themes based on user-perception studies, such as user’s perceptions on positive and negative issues, perceived benefits, and opinions on removing the system. This approach allowed us to delve deeper beyond standardised variables into users’ candid perceptions.

The data were collected between February and September 2011. In terms of sampling, we obtained a list of pharmacies classified as early adopters from CfH and selected pharmacies that were ‘live’ and dispensing above 5% electronic prescriptions. The eight pharmacies that fell into this category were in the midland and northern regions of England (Table 1). Sites with different computerised pharmacy management systems were chosen to get divergent perspectives. Other variations in the sites sampled included type of ownership of the pharmacy (independent or chain), geographic location and different software suppliers. Due to the small number of test sites (initial adopters) at the time of the study, providing detailed information in this paper would breech confidentiality and commercial sensitivity agreements. Permissions were sought from approval bodies at each of the study sites, including Primary Care Trusts, research governance teams, benefit realisation teams and informatics leads. The study protocol was submitted to the Cambridgeshire Research Ethics Committee who classed the study as a service evaluation.

Data were collected using ethnographically informed mixed methods including observations, formal (recorded) and informal interviews, and shadowing of pharmacy professionals. Our chief participants were pharmacists/pharmacist proprietors as they had overall insight of the EPS2 integration into work practice. We conducted audio-recorded interviews with all chief participants. We also audio-recorded one accredited checking technician and one dispenser as additional participants. We conducted 10 audio-recorded in-depth interviews. The interviews took 30 minutes to one hour, with transcripts for each averaging 3700 words. Notes were recorded from the participants too occupied with work to give audio-recorded interviews. While the pharmacists were generous in allowing us access to their sites and permitting interviews, the busy nature of the job meant there were time constraints.

### Analysis

**Figure 2:**
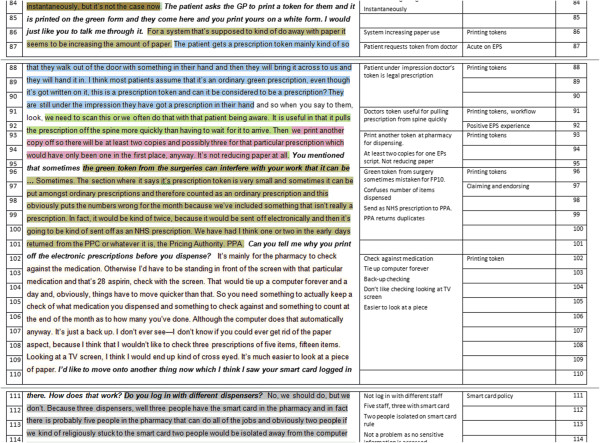
Sample line-by-line coding method.

Overall, 37, 200 words from the transcripts were analysed besides the field notes. Both types of qualitative data were analysed using line-by-line coding (Figure 2) and thematic analysis. Each line was coded into subthemes and into themes. Themes were then recoded into positive and negative issues. This identified experiences associated with usability and user experiences thereby achieving a bottom-up analysis. EPS is a standardised system that has to be integrated with different pharmacy dispensing systems, meaning CPs have a choice of who their ‘pharmacy system-EPS’ supplier is [31]. The eight sites had different pharmacy system suppliers. Bearing this in mind, we focused our analysis on common issues among the sites to remove supplier specific issues.

Analysis showed that CP professionals were overall positive about the system and wished for it to be retained and improved, instead of being discontinued [32]. They were however facing two types of challenges with the system. The first type of challenge was caused by missing electronic prescriptions. Sometimes, when the prescriber wrote the prescription, pharmacists nominated by the patient to dispense the prescription could not see or download the prescription for dispensing. This issue was especially challenging for pharmacists as there were different causes with no adaptable solution within pharmacy work practice. Pharmacists, however, considered this a teething issue that could be resolved by small modifications to the system [32]. The second type of challenge was considered long-term and was specific to the system design. Although these design specific issues were adaptable into work practice with ‘add-on’ designs, pharmacists thought these issues were present because they were not involved in the system design and development. We decided to present the two challenges in two papers to allow us to discuss the issues (with user commentaries) in detail. In this paper, we present our set of second type challenge in the EPS2 adoption.

## Results

We found there were some essential user needs that were not met. These caused users to interact with the system in ways not intended by the system developers, and interfered with how CP professionals experienced the system. These were, 1) dispensing from printed-out tokens instead of screens, 2) using one Smartcard to log all dispensing activities by different staff, and, 3) problematic interface for claiming reimbursements.

### Printing tokens

**Figure 3:**
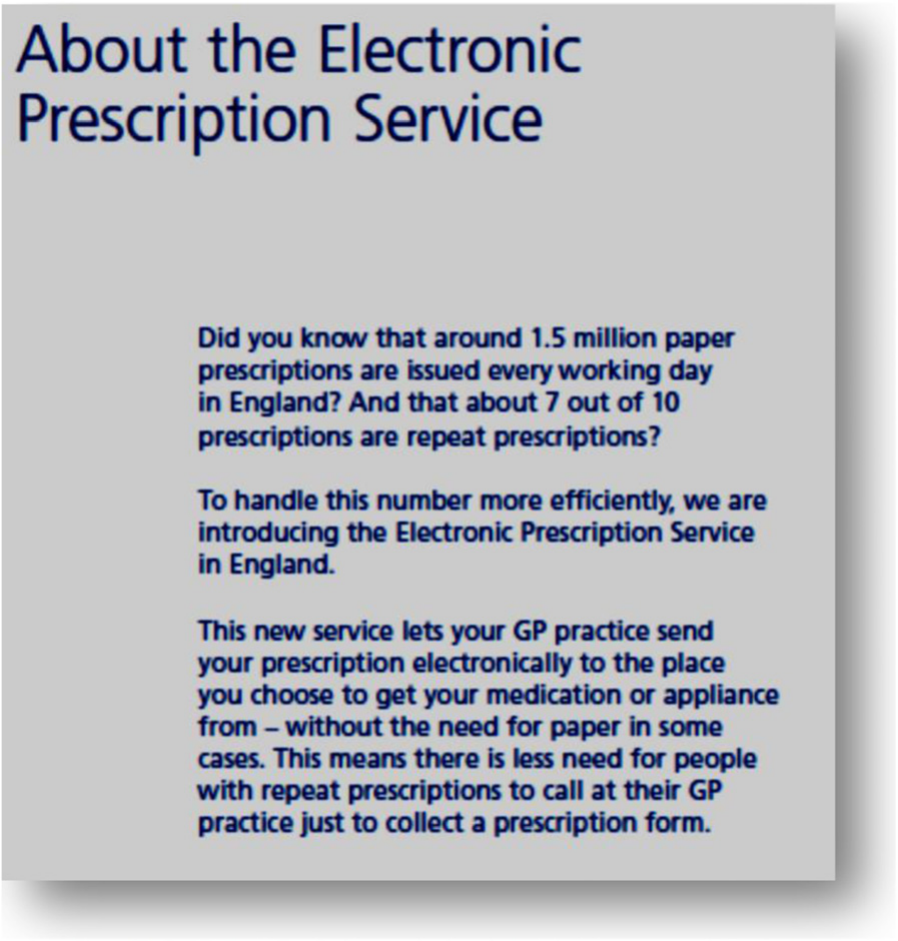
NHS EPS2 patient and carers information leaflet in England.

A key aspect of the EPS2 design was to decrease paper prescriptions by electronically writing and dispensing prescriptions. Figure 3 shows how the system was described in the patient and carer information leaflet [33]. This means prescribers have to record the medications electronically on their computer screens and dispensers, after downloading the prescription, can dispense them directly from the screen without any need of a paper. The pharmacists we interviewed would not dispense from a computer screen, as they feared it would compromise safety. They felt that the best practice was to have a paper prescription beside the medicines they were dispensing. This enabled dispensers to check medications they were picking from the shelves against the paper in hand, and allowed pharmacists to conduct the final professional check of the dispensed items from any location in the pharmacy. Pharmacists felt that dispensing from computer screens would not only hinder this form of verification but restrict access to the computers as the following excerpts demonstrate:

*I don’t know whether their initial aim was to dispense off the screen or check off the screen, you know, but I wouldn’t check off the screen or dispense off the screen at all. I need a piece of paper in front of me, so that defeats the purpose of the electronic prescriptions*” (Site 3, Pharmacist).

“*Often, you are too busy in a pharmacy to be able to dispense from the screen. You might have to run your labels off and you need something to check from*” (Site 1, Pharmacist).

“*I don’t ever see—I don’t know if you could ever get rid of the paper aspect, because I think that I wouldn’t like to check three prescriptions of five items, fifteen items looking at a TV screen. I think I would end up kind of cross-eyed. It’s much easier to look at a piece of paper*” (Site 8, Pharmacist).

CPs felt they would have made this known had they been consulted in the EPS2 design. Consequently, instead of directly dispensing medication from computer screens, community pharmacists have deviated from the original EPS2 design by opting to print out the electronic prescriptions after downloading them onto their screens. These printed electronic prescriptions, commonly known as tokens, were then used to dispense medications. Although tokens are not legal prescriptions, they provide a hard copy of data from the electronic prescription, so were accepted by pharmacists for use in dispensing. In addition, printing the tokens enabled patients to have a physical possession of their medication information, as explained by this pharmacist:

“*You might need the token to be able to check your items to the labels. Also, you need the repeat slip for patients attached to the token. Patients aren’t going to be able to order again unless they get something in their bag and they can order next time. To do that you need to print the token. If the patient is exempt in some way or pays for the prescription, it has to be filled in on the back if they are not age exempt. If they are under 16, or, over 60, you don’t have to use that paper copy. However you still need the repeat slips, you still need to fill in the exemption and get it signed on the back to show that the patient has an exemption or has a certificate to show that they don’t pay*” (Site 1, Pharmacist).

On 10 October 2012, E-health Insider (EHI) featured an article that showed plans for the NHS to be paperless by 2015 [34]. From these interviewee commentaries, it is apparent that the paper-free design of EPS2 is challenging to its users in practice. Paper prescriptions are small, light and mobile and can be annotated, for example, in the final safety check the pharmacist can tick each item on the paper as it has been confirmed correct, allowing them to be interrupted and regain their place without losing accuracy. Perhaps other ‘paper-free’ alternatives could be explored such as using tablets to take screenshots of the electronic prescription for portable dispensing. A challenge in relation to printing of tokens is the additional cost; that pharmacists take this on without reimbursement shows the printing has value to them.

### Smartcard policies

EPS2 was designed to be a Smartcard system to enable access to the Spine. Health professionals who need access to the Spine have to apply for Smartcards from the Registration Authority usually the local Primary Care Trust (PCT) [3]. Since EPS was designed to use ‘a single card’ model of access, individual Smartcards are required for each user. Users cannot share Smartcards or even share access sessions. The purpose of this is to have an audit trail of users of the Spine [3,35]. Generally, the Smartcard system was not used as intended. Community pharmacies are places of constant rapid physical acts fulfilling the prescription; time at a terminal is a relatively small part of this and terminals are often used by several people. CPs felt that this was the more practical usable method since multiple users did not have to keep logging in and out of the system. Consequently, the first person who logged into the system left their Smartcard in until they finished work for the day then someone else would take over as the main logged-in user as the following excerpt demonstrates:

“*We do all have individual Smartcards. We all can use an individual Smartcard. It would just tend to be whoever has been logged in first thing in the morning as the person who would end up doing that most of the day. S goes home at half past four, for instance, and then someone else would have to do it. SE would probably log-in, she leaves at six and then whoever is in this evening would log-in. It just depends really as to who is in first thing in the morning. But yeah, that’s just the way it goes*” (Site 5, Pharmacist).

The main reason behind this approach was convenience as the logging in and out by different users would significantly interfere with workflow and reduce productivity, especially at peak hours. Some pharmacists also argued that the reason behind using one Smartcard was to distribute the workload as widely as possible. In the following, pharmacists give more explanation of their reasons for not using Smartcards for each member of staff:

“*No, we should do [log-in individually], but we don’t. Because three dispensers, well three people, have the Smartcard in the pharmacy and in fact there is probably five people in the pharmacy that can do all of the jobs and if we kind of religiously stuck to the Smartcard [rule], two people would be isolated away from the computer not being able to dispense. I log-in at the beginning of the day and it’s left there. My responsibility if anything happens untoward, but I can’t understand what it could be, because they are doing NHS prescriptions all the time anyway, and they are used to doing that, and they’ve signed the Data Confidentiality and Data Protection Act*” (Site 8, Pharmacist).

“*We keep our own. We’ve [each] got a screen, a monitor each, maybe because there is only two of us so X keeps her Smartcard and if I’m not in and if there is a locum in, X will download all the prescriptions, because well, our regular locum does have her own Smartcard and she will bring that and use that. But if you use an agency locums, they wouldn’t necessarily have a card and then everything would go through X”* (Site 6, Pharmacist).

Clearly, this was not how the system was designed, continued use this way could breach security and might become an issue if pharmacists have access to patients’ electronic health records. Since EPS2 is still undergoing product development through modifications, perhaps this feature could be modified in a way that would not require the logging of every user’s activity.

### Prescription endorsing interface

Getting the interface right in any system development is often the biggest challenge. The interface of a system is a self-contained feature and includes getting a range of multiple items right such as the aesthetics, clarity in direction of use, colours, and user’s emotional connection with it [36]. A key interface issue that has arisen at the evaluation stage of the EPS2 is interacting effectively with the endorsing and claiming interface. When prescriptions are dispensed, pharmacists have to send off the prescription details to NHS prescription service, which has several functions including reimbursing the price of the purchased medicines and the professional fees incurred by pharmacists. Under the paper system, the prescriptions are collated and sent off manually to the pricing body; under the EPS2, this is done electronically using the ‘endorsing and claiming’ interface. The problem some pharmacists appeared to be having with EPS2 interface is effectively interacting with it to claim back charges incurred after dispensing. Since a Common User Interface (CUI) that would integrate with different types of pharmacy systems was not developed [37], some pharmacy systems adapted better to endorsing and claiming than others. Consequently, some pharmacists had more issues than others. However, a common concern involved ‘NCSO’ claiming, as this pharmacist explains in detail:

“*NCSO means No Cheaper Stock Obtainable. Normally everything gets endorsed by the computer, by the endorsing machine. Certain items have to be endorsed manually [not automatic but completed on screen] if it’s specialised and controlled drugs or NCSO. Every month, the Department of Health, they produce a list. Sometimes it’s quite a big list, ten, fifteen items and sometimes it’s only one or two items. For example, this month, 20 mg tablets is on the NCSO list. That list only gets published about the tenth of each month. Everything I’ve done from the first to the tenth, I don’t know whether that item is going to be on the NCSO list. With NCSO, what I have to do, I have to sign it, and I have to date it and I have to put the price I’ve paid for it. So the (Inaudible 00.12.22) might be £2.50 for example. What I’ve paid for it is £10. If I can’t endorse it as £10 I lose £7.50.”* (Site 3, Pharmacist).

The encouragement by NHS Connecting for Health of pharmacists to regularly claim electronically for reimbursement (to reduce high flows at the end of the month) means that they may not get reimbursed for NCSO list items that they have dispensed and sent off for reimbursement early in the month. This issue has led pharmacists to become concerned about losing income as noted in the following extracts:

“*We are still having problems transmitting a lot of data to pricing bureau. Last month, I think we had about two dozen in total that wouldn’t allow them to be electronically submitted. Our main problem is, when you come to do your ETPII endorsing on [software system 2], I think it was the same on [software system 1]. If you supply, say aspirin tablets and you say aspirin tablets and it has a manufacturer next to it Tabba Actevis. What we’ve done is, we’ve obviously ticked the NCSO button. Because we’ve logged in, we assume that’s as good as a signature for them, which it is, apparently and we sent the data off for months and months and months. We haven’t been paid for any NCSO for months*” (Site 2, Pharmacist Proprietor).

“*When we endorse, often there is prescriptions that just won’t go. They won’t send so we’ve [wrongly] paid for numerous prescriptions where I’m going to have to sit down with X from the PCT [Primary Care Trust] next week and go through them all and see whether we can chase these payments. Some of them are over six months old, so then we’d have to request the doctors to reissue. One of the endorsing issues was for PIs imports that should get paid the same as a normal English pack. The C programme was asking for a supplementary payment, which is not required for this item. But because I’ve spoken to pharmacists who work in a pharmacy and obviously [they’ve] spoken to a pharmacist who has been stuck in their computer room for the last ten years, they don’t realise what’s going on. They basically said to us, well, for it to go you need to put a payment in or a supplementary payment. They said, well, just put a penny in and they will pay you for the right amount. Some of these cost a lot of money. I said well, I tell you what then, if they are going to pay the right amount, why should I put a penny in and risk it. I’ll put £10,000 in shall I, and then I will get paid still the right amount and not the £10,000. If it goes wrong, I’m at least on the right side of it. So then I get the PPA ringing me up and wondering why I’ve endorsed a load of prescriptions for millions of pounds and then again I’m under stress again, because the PPA are accusing me then of trying to claim lots of money, I was just trying to prove a point”* (Site 1, Pharmacist Proprietor).

These detailed commentaries define an interface problem that appears to be causing monetary stress to some pharmacists. Since the interface was not user-friendly, users had to look for alternative ways to claim reimbursements of NCSO items.

## Discussion

### Usability and user experience: meeting user needs

In our findings, CP professionals’ needs were not met, particularly in how the system was designed so they had to find ways to adapt them to their needs. The usability and user experience issues identified did not conform with key facilitators of technology adoption such as ease of use and compatibility [9,38]. While an explanation of the unintended uses could be attributed to CPs collective resistance to change, it does not appear as if the system was tailored to their needs in the first place, which is a key facilitator of technology adoption. A different explanation to these design specific issues could be due to the lack of CPs’ perspectives in the design and development of the EPS2, as demonstrated by this comment: “*But because I’ve spoken to pharmacists who work in a pharmacy and obviously [they’ve] spoken to a pharmacist who has been stuck in their computer room for the last ten years, they don’t realise what’s going on”.* Whether or not this statement is justified, in the following, we use the concept of user-centric principles to explore and discuss how the involvement of CPs as a key user group in the EPS2 development might have enhanced their experience.

### User-centric approaches and active user involvement

To meet user needs in terms of system design, users are often involved in the system development process [38]. Applying user-centric principles, such as user-centred design, which moulds the design of the system to suit its intended users, is recognised as best practice [39]. Studies have shown that national healthcare Information and Communication Technologies (ICTs) development such as Electronic Health Records (EHRs) tend to fail in user engagement [19]. A key reason for this is, users tend to be excluded from the initial stages, and are usually involved only at later stages, thereby making critical modifications to the system design either expensive or impossible [40-42].

Involving users in a new system development can take several approaches. While active involvement of users is sometimes seen as overindulging by allowing users to specify unnecessary functions [43], the application of user-centric design is usually seen and adopted as best practice in a system development. User-centred design, sometimes also referred to as ‘user-centred system design’ is a human-computer interaction strategy to involve end-users in a new system or service development. The meaning of UCD has become vague since it was coined by Norman and Draper [44], however, its aim is to place emphasis on user engagement, and improving how the user experiences the system when implemented. It focuses on users by learning about the context of their work, the environments they work in, and their needs for usable products [45]. In short, the aim of UCD is to create a better user experience for the people for whom the system was designed. In practice, UCD faces many challenges as it can be applied using several approaches [39,45]. Using common approaches to UCD, Gulliksen et al. [39] developed key principles that underpin its application in a system development, and these are:

• The early focus on users’ work practices and tasks to control the development,

• Active user participation in the analysis, design, development and evaluation,

• Early prototyping to gradually build a shared understanding of user needs as well as future work practices,

• Continuous iteration of designed solutions,

• Multidisciplinary design teams, and

• Integrated design that involves the system, the work practices, online-help, training, organisation, etc.

The theory about the application of these principles is that they will improve the usability of the product and its User Interface (UI) such as ease of use, efficiency, reduced error, and user satisfaction. UI has especially become a current focus of UCD practices as it enables users to familiarise themselves with the product’s interface early [46]. When detailed iteration takes place in the later stages of the development, it is more expensive if the design follows a stage-gate model from conceptual to detailed design. Meaning each design step sets constraints on the next and hence any change in design at a later stage is more expensive than at an earlier stage [47]. Using this perspective, we put into context how a UCD approach could have been used to mould CP professionals’ experience of EPS2.

### Putting user-centeredness into context

While reasons such as lack of technical and other forms of support, no added value in terms of finance, or even resistance to change could be explanations for why CPs could not interact effectively with the design of EPS2 [9,20,38], active user-involvement could be another explanation. Firstly, in terms of dispensing directly from screens, CPs elected to print and dispense from tokens because various usability requirements such as equipment sharing, job sharing, multi-tasking, and providing advisory slips to patients made dispensing directly from screens challenging. This issue was evident during the piloting of various models at the concept evaluation stage [1]. However, it appears the issue was not explored further or resolved before latter stages, as it would have also been evident during the prototype testing stage. Secondly, in terms of CPs electing to use a single smartcard for multiple users, a key practice of UCD is to perform detailed task analysis of users’ work to design systems that would improve (not interfere) with workflow [45]. Using multiple user log-ins to achieve accurate audit trails appears a feasible concept. In practice however, the concept was challenging for CP professionals as it interfered with workflow. They therefore adapted the Smartcard’s usability to suit their needs by using one Smartcard to serve multiple users. This issue was evident in our EPS2 pre-implementation study of pharmacy work practice as pharmacists showed signs of not using EPS2 Smartcards as intended. We fed back our findings to participating PCTs as part of our formative evaluation [48]. However, this issue could have been evident much earlier at the concept evaluation stage when the idea of EPS2 (and this function) was being marketed to users. It could also have been identified during the user’s task analysis or even the prototyping stages of user-centric approaches. Lastly, since a common user interface to integrate with different types of pharmacy managing systems was not developed, pharmacists had to find ways to deal with the NCSO claiming and endorsing function. Prototype testing by users from various dispensing systems could have aided a better NCSO UI design across all systems.

We do not know to what extent user-centric approaches were used and how rigorously they were applied as we could find very little information on this. It is possible that these design issues were identified at earlier stages but were not resolved at later stages of the system development, however, the purpose of active user involvement is to mould the system to user needs before it becomes too expensive to resolve [47]. While, there may be other explanations for why users could not integrate these EPS2 features into work practice, our study suggests that lack of active CP user groups in the early stages of system development may have been the main problem. While engaging users at later stages allows them to touch and feel the actual product and not just a concept, active involvement in the initial stages is more participatory and allows users to contribute to the design based on their needs. The EPS2 at its evaluation stage clearly showed some important technical barriers that could have perhaps been eliminated had real users been meticulously and actively involved in the initial stages of its development.

### Strengths

We have built on work done by Cresswell et al. [19] on user engagement by focusing on community pharmacists and their teams as a key user as key user group in the delivery of the national Electronic Prescription Service (EPS) in England. Our focus on English community pharmacies (and primary care) in this paper contributes new knowledge because most of the user-perception literature on national electronic prescriptions systems relates to other parts of Europe and America, and focuses on general practice (GP) or secondary care [20,24-29].

### Limitations

We emphasize that these are only preliminary findings from early adopter sites, and suggest further studies to establish how the system operates when more mature. We acknowledge that with a small sample our findings may not be generalisable to other community pharmacies, or fit with the experiences of pharmacies that adopted EPS later. However, our aim is to draw attention to involving user group perspectives into systems’ development regardless of whether the issues were still persistent at the time of writing this article. Overall, there is scope for further studies once EPS2 has been implemented more widely. This could help determine whether issues we have identified have been resolved and/or whether new issues have emerged.

## Conclusions

Using detailed commentaries from the study of early adopters, we have drawn attention to some key issues in national healthcare system development from a community pharmacy perspective. Firstly, pharmacy professionals needs were not meet in terms of the system design as they had to appropriate certain aspects of the system to suit their needs. Secondly, the process of involving users should be made publicly available to reassure users and to make the development process transparent. Thirdly, our findings suggest that involving real users in the service or system design from the initial stages and throughout the development life cycle could help enhance usability and user experiences, or at least flag up cases in which national policy will be detrimental to local activities. While we acknowledge that many pharmacists were over all in favour of the system, EPS2 at this early evaluation stage clearly showed some key usability and user experience barriers that could have perhaps been eliminated had real users been meticulously and actively involved in the initial stages of its development. We conclude that since EPS2 at the writing of this article is in its evaluation stage, these issues can still be addressed through modifications and perhaps system redesign, and would help meet the needs of community pharmacies and improve their user experience.

### Recommendations

Our recommendations take into account that the EPS is still evolving, and therefore need long term (or futuristic) solutions to the design challenges faced by pharmacists, if a paperless NHS is to be achieved.

1. Paperless alternatives to the prescription should be developed and assessed with users.

2. Alternative governance arrangements, or different technology (such as biometric identification), is needed to allow several different users to use the same terminals without delay.

3. In terms of interface challenges of claiming and reimbursement, pharmacists could be encouraged to make NCSO claims, after the list is published by the Department of Health, while EPS is evolving. In the long term, we recommend that pharmacists make their collective voice heard regarding this issue so that a more acceptable ‘standardised’ interface could be achieved.

4. Pharmacists should form powerful user groups to work with each of the pharmacy management system suppliers.

## Competing interests

The authors declare they have not competing interests.

## Authors’ contributions

JH facilitated the refinement of the research design, was the primary data collector of the study. She conducted the in-depth interviews and recorded the nonparticipant observations field notes from all eight early adopter pharmacies. JH conducted the analysis and interpretation of the data with AA and NB and other members of the team, and led the writing of this paper. AA co-authored and edited all the cases studies from the study, extensively contributed to the analysis and the interpretation and of the data and provided commentary on the writing up process. RH provided insights into user group involvement in the EPS2 planning and development by interviewing the relevant technical architects and making available user group meeting minutes. RH also provided constructive feedback on this paper. NB contributed to the analysis and critical interpretation of the data analysis and provided key insights to improve the paper. All authors read and approved the final manuscript.

## Authors’ information

JH is a social scientist and specialises in Social Informatics, in particular, ethnological study of peoples’ (dis)engagement with technologies. She has a multidisciplinary background in human sciences, information science and information technology. She works with Professor Tony Avery at the School of Medicine, University of Nottingham. JH previously worked at the Department of Social Sciences, Loughborough University, and WMG at the University of Warwick.

AA is professor of Primary Health Care at the Division of Primary Care, School of Medicine, University of Nottingham. Among other interests, he specialises in patient safety and the use of information technology to aid clinical practice, and has an extensive portfolio and high starred academic papers for both quantitative and qualitative research in this field. AA is also an active general practitioner in the city of Nottingham.

RH was a Research Fellow at the Department of Practice and Policy, University College London School of Pharmacy, University of London. RH has a background in technical design and engineering. His position as the lead researcher of the EPS2 project enabled him to gain critical insight into EPS2 development as he has access to implementer and user group meetings and, liaised closely with Connecting for Health.

NB is professor of the Practice of Pharmacy at the Department of Practice and Policy, University College London School of Pharmacy, University of London. NB gave a critical insight throughout the research write up. NB is also a visiting professor of medication safety at Harvard Medical School and is a practising pharmacist.

## Pre-publication history

The pre-publication history for this paper can be accessed here:

http://www.biomedcentral.com/1472-6947/14/16/prepub
